# Using Eddy Covariance Sensors to Quantify Carbon Metabolism of Peatlands: A Case Study in Turkey

**DOI:** 10.3390/s110100522

**Published:** 2011-01-06

**Authors:** Fatih Evrendilek, Nusret Karakaya, Guler Aslan, Can Ertekin

**Affiliations:** 1 Department of Environmental Engineering, Abant Izzet Baysal University, 14280 Golkoy Campus Bolu, Turkey; E-Mails: karakaya_n@ibu.edu.tr (N.K.); aslan_g@ibu.edu.tr (G.A.); 2 Faculty of Agricultural Engineering, Akdeniz University, Antalya, Turkey; E-Mail: ertekin@akdeniz.edu.tr (C.E.)

**Keywords:** carbon cycle, flux tower, biogeochemical model, diurnal variation

## Abstract

Net ecosystem exchange (NEE) of carbon dioxide (CO_2_) was measured in a cool temperate peatland in northwestern Turkey on a continuous basis using eddy covariance (EC) sensors and multiple (non-)linear regression-M(N)LR-models. Our results showed that hourly NEE varied between −1.26 and 1.06 mg CO_2_ m^−2^ s^−1^, with a mean value of 0.11 mg CO_2_ m^−2^ s^−1^. Nighttime ecosystem respiration (*R*_E_) was on average measured as 0.23 ± 0.09 mg CO_2_ m^−2^ s^−1^. Two best-fit M(N)LR models estimated daytime *R*_E_ as 0.64 ± 0.31 and 0.24 ± 0.05 mg CO_2_ m^−2^ s^−1^. Total *R*_E_ as the sum of nighttime and daytime *R*_E_ ranged from 0.47 to 0.87 mg CO_2_ m^−2^ s^−1^, thus yielding estimates of gross primary productivity (GPP) at −0.35 ± 0.18 and −0.74 ± 0.43 mg CO_2_ m^−2^ s^−1^. Use of EC sensors and M(N)LR models is one of the most direct ways to quantify turbulent CO_2_ exchanges among the soil, vegetation and atmosphere within the atmospheric boundary layer, as well as source and sink behaviors of ecosystems.

## 1. Introduction

Though spatially small (5% of the terrestrial biosphere) compared with most other ecosystems [1,2], peatlands play a significant role in carbon (C) and water metabolism of the World. Understanding and quantifying C dynamics of peatlands are crucial to prediction of responses to global climate change and rehabilitation of peatlands under the increasing magnitude and rate of human-induced disturbances. Eddy Covariance (EC) sensors are one of the most direct ways to measure and estimate turbulent carbon dioxide (CO_2_), water vapor and energy fluxes exchanged among soil, vegetation, and atmosphere within the atmospheric boundary layer. The use of the EC method and sensors on a long-term and continuous basis across the World has led to the establishment of an integrated global network for standardization of flux tower activities (called FLUXNET) and a network for standardization and development of spectral sensors toward bridging the gap between remote and proximal sensing (called SpecNET).

Our study area in Turkey, the Yenicaga peatland area that at one time occupied 240 km^2^, has diminished to less than 30 km^2^ due to drainage, cultivation, afforestation, and peat mining [3,4]. There is a lack of information about C metabolism of peatland ecosystems in Turkey, and this study is the first to comprehensively determine C dynamics and components in one of the remaining major peatlands. The objective of this study was to quantify the rate, magnitude, and timing of CO_2_ exchange between the atmosphere and Yenicaga peatland using the EC sensors and multiple (non-)linear regression-M(N)LR-models.

## 2. Study Site and Methodology

### 2.1. Description of Study Site

**Figure 1.**
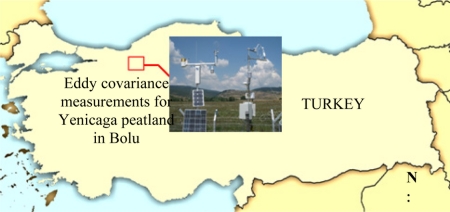
Location of the study site “Yenicaga peatland” in northwestern Turkey.

The Yenicaga peatland is located about 38 km east of the city of Bolu (40°47N′, 32°1′E) in the western Black Sea region of Turkey (Figure 1).

An EC flux tower site was installed about 1 km north of Lake Yenicaga (18 km^2^) at the elevation of 988 m above seal level on July 12, 2010. The climate in the Yenicaga region is classified as cool temperate, with a mean annual temperature and precipitation of 10.2 °C and 538 mm, respectively [5]. The Yenicaga peatland is reported to contain Devonian and cretaceous limestone, basaltic tuff, lava, and olistolites, with the uppermost layer consisting of tertiary and quaternary formations [5]. The natural vegetation types of the Yenicaga peatland consist of the following dominant communities: (1) *Phragmites australis* and *Typha domingensis* (41.2 ha); and (2) *Ranunculus lingua, Acorus calamus, Najas marina, Pedicularis palustris, Senecio paludosus* (107.8 ha) [6]. The mean vegetation height around the flux tower is about 0.5 m, and the terrain observed around the flux tower exhibits flat and uniform grasslands.

### 2.2. Eddy Covariance Flux and Ancillary Measurements

Net ecosystem exchange (NEE) rates of carbon dioxide (*F*_c_, mg m^−2^ s^−1^) in Yenicaga peatland were estimated using an eddy covariance (EC) system consisting of an open-path CO_2_/H_2_O gas analyzer (LI-7500, Licor Inc., Lincoln, NB, USA), a 3-D sonic anemometer/thermometer (CSAT3, Campbell Scientific Inc., Logan, UT, USA), a data logger (CR3000, Campbell Scientific Inc.), and a 3-m tower on which EC flux sensors were mounted. The distance between the LI-7500 and CSAT3 sensors was 0.15 m, with CSAT3 oriented towards the prevailing wind direction (an azimuth angle of 30° from true north) and LI-7500 vertically rotated 15° towards the footprint.

Eddy fluxes and associated signals were recorded at 10 Hz, block averaged over one hour (h) and corrected for the effects of fluctuations in air density on CO_2_/H_2_O fluxes (*F*_c_wpl_ and LE_wpl_ with WPL correction) through the online flux computation. EC data were collected swapping two 2-GB Compact Flash cards at 14-to-18-day intervals. The net radiation (*R*_n_), downwelling and upwelling longwave (4 to 50 *μ*m) and shortwave radiation (0.2 to 4 *μ*m) (*R*_l_dn_, *R*_l_up_, *R*_s_dn_, and *R*_s_up_, W m^−2^) were measured using Kipp & Zonen CNR-4 net radiometers (Kipp & Zonen USA Inc., Bohemia, NY, USA). Air temperature (*T*_a_, °C), and relative humidity (RH, %) were sampled using HMP45C probe (Vaisala, Finland). Precipitation (PPT, mm), evapotranspiration (ET, mm), soil water content (SWC, %), and mean, maximum and minimum soil temperature (ST, ST_max_, and ST_min_, °C) were measured on a hourly basis using ET107 weather monitoring station (Campbell Scientific Inc.). Photosynthetically active radiation (PAR) was estimated from net shortwave radiation (*R*_s_n_) using a conversion factor of PAR: *R*_s_n_ = 0.5 [7–9]. The values of CO_2_ fluxes in unit of mg m^−2^ s^−1^ were also converted to unit of kg C ha^−1^day^−1^, based on the following conversion ratios of mg:kg = 10^6^; s:day = 86,400; m^2^:ha = 10,000; and CO_2_:C = 44/12.

### 2.3. Data Processing and Analyses

As with the conventional meteorological sign notation, downward and upward fluxes are considered negative (−) and positive (±), respectively. The first step of data processing involved the removal of erroneous spikes and their associated CO_2_ fluxes when latent heat flux (LE_wpl_) < −100 or > 800 W m^−2^; sensible heat flux (*H*_s_) < −150 or > 500 W m^−2^; and precipitation events occurred [10]. For both hourly daytime and nighttime CO_2_ fluxes, descriptive statistics were given, and best-fit cumulative distribution function (CDF) was selected. Tukey’s multiple comparison was performed after one-way analysis of variance (ANOVA) for the entire dataset in order to test significant differences in hourly, nighttime *versus* daytime and monthly means. All the statistical analyses were performed with Minitab 15.1 (Minitab Inc. 2006).

Second, EC data were separated into daytime (*R*_n_ and/or *R*_s_dn_ > 10 W m^−2^) and nighttime (*R*_n_ and/or *R*_s_dn_ ≤ 10 W m^−2^) periods since EC data are more reliable during daytime hours than during nighttime hours, and nighttime EC data ( $F_{c\_\text{wpl}}^{\mathrm{night}}$) can be used to estimate daytime as well as nighttime ecosystem respiration (both plant and soil respiration) ( $R_{E}=F_{c\_\text{wpl}}^{\mathrm{night}}$). Negative night CO_2_ flux data were deleted as no gross primary productivity (GPP = 0) occurs during the nighttime. The site-specific threshold value of friction velocity (u*) was determined as 0.03 m s^−1^ below which low vertical wind velocity led to underestimation of the nighttime CO_2_ fluxes [11]. Likewise, nighttime periods where horizontal wind velocity was less than 1 m s^−1^ were removed from the dataset. Finally, night fluxes where CO_2_ density data had the standard deviation of > 14 mg m^−3^ (0.6 μmole m^−3^) were eliminated from further analyses [12]. Multiple (non-)linear regression models were fitted to the resultant night CO_2_ dataset, and best-fit M(N)LR models with and without the inclusion of temporal variables (hour, and month) were chosen using best subsets procedure (low Mallows’ *C*_p_, high adjusted *R*^2^, and low SE). The following soil respiration equations of RothC [13] and CENTURY [14] models were also used to model daytime *R*_E_:

(1) $$f(R_{\text{soil}})=\frac{47.9}{1+\text{exp}\;\left(\frac{106}{\text{ST}+18.3}\right)}\text{for RothC model}$$

(2) $$f\left(R_{\text{soil}}\right)=0.56\pm 0.465^{*}\text{arctan}(0.097^{*}(\text{ST-}15.7))\;\text{for CENTURY model}$$

where *R*_soil_ is soil respiration (mg CO_2_ m^−2^ s^−1^), and ST is soil temperature (°C). Net ecosystem exchange (NEE, mg CO_2_ m^−2^ s^−1^) can be expressed as follows:

(3) $$\text{NEE}=-\text{GPP}\pm R_{E}$$

Hourly GPP values were estimated as the sum of NEE and daytime *R*_E_. Daytime *R*_E_ was obtained extrapolating nighttime *R*_E_ fluxes to the remaining daytime fluxes, based on M(N)LR models.

## 3. Results and Discussion

### 3.1. Diurnal CO_2_ Fluxes

**Figure 2.**
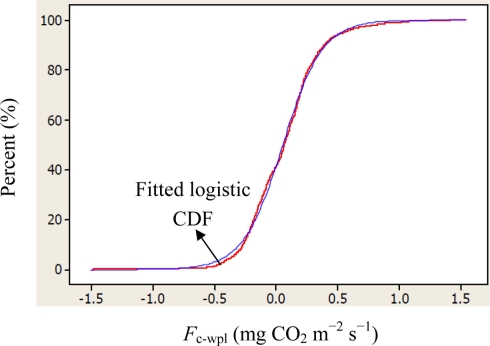
Logistic cumulative distribution function (CDF) fitted to both daytime and nighttime CO_2_ fluxes for the Yenicaga peatland: (Location (μ) = 0.05197; scale (*s*) = 0.1596; *r* = 0.99; *n* = 1884; *P* < 0.001).

Descriptive statistics of despiked and averaged EC data for the period of July 12 to October 17, 2010 indicated that mean hourly CO_2_ fluxes above the canopy had a larger temporal variability (CV = 498) than the rest of the measured variables and varied between −1.5 and 1.5 mg m^−2^ s^−1^ (Table 1). During the study period, the study site cumulatively received 92 mm PPT and lost 305 mm water vapor via ET. The mean Bowen ratio of *H*_s_:LE_wpl_ quantifying the evaporative demand of an environment was estimated at 0.37, thus characterizing the Yenicaga peatland as a mesic environment. A logistic CDF with a location of 0.05197 and a scale of 0.1596 appeared to fit our daytime and nighttime *F*_c_wpl_ data fairly well as follows (*r* = 0.99; *n* = 1884; *P* < 0.001) (Figure 2):

(4) $${\text{CDF}}_{\text{logisitic}}=\frac{1}{1+e^{-(F_{c-\mathrm{wpl}}-\mu )/s}}$$

where *μ* and s are location and scale parameters of the logistic CDF, respectively. The fitted logistic CDF can be used to estimate percentiles for the CO_2_ flux data (Figure 2).

**Figure 3.**
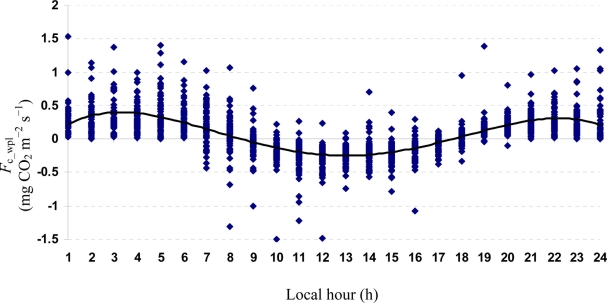
A quartic function fitted to the entire *F*_c_wpl_ dataset in the Yenicaga peatland for the period of July 12 to October 17, 2010: *F*_c_wpl_ (mg CO_2_ m^−2^ s^−1^) = 0.2807 h − 0.0602 h^2^ ± 0.0038 h^3^ − 7 × 10^−5^ h^4^; (*R*^2^_adj_ = 0.5368; SE: 0.2016; *n* = 1884; *P* < 0.001).

**Figure 4.**
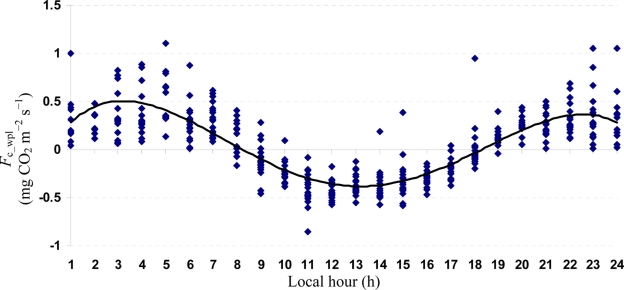
A quartic function fitted to *F*_c_wpl_ dataset for Yenicaga peatland in July of 2010: *F*_c_wpl_ (mg CO_2_ m^−2^ s^−1^) = 0.3575 h – 0.077 h^2^ ± 0.0049 h^3^ – 9 × 10^−5^ h^4^; (*R*^2^_adj_ = 0.7087; SE = 0.1905; *n* = 412; *P* < 0.001).

**Figure 5.**
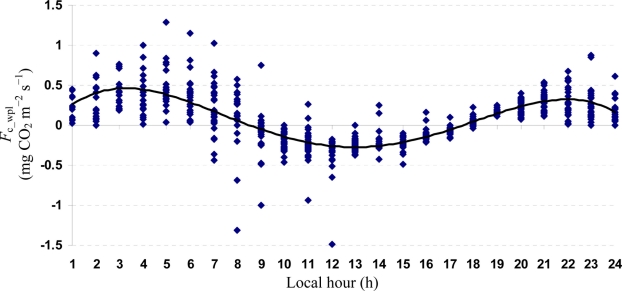
A quartic function fitted to *F*_c_wpl_ dataset for Yenicaga peatland in August of 2010: *F*_c_wpl_ (mg CO_2_ m^−2^ s^−1^) = 0.3242 h − 0.0696 h^2^ ± 0.0044 h^3^ – 9 × 10^−5^ h^4^; (*R*^2^_adj_ = 0.5963; SE = 0.1957; *n* = 620; *P* < 0.001).

**Figure 6.**
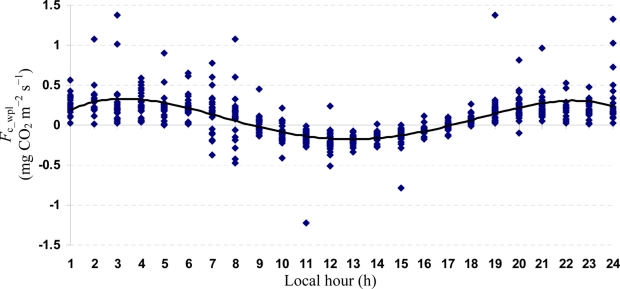
A quartic function fitted to *F*_c_wpl_ dataset for Yenicaga peatland in September of 2010: *F*_c_wpl_ (mg CO_2_ m^−2^ s^−1^) = 0.2297 h − 0.0488 h^2^ ± 0.0031 h^3^ – 6 × 10^−5^ h^4^; (*R*^2^_adj_ = 0.4726; SE = 0.1875; *n* = 603; *P* < 0.001).

**Figure 7.**
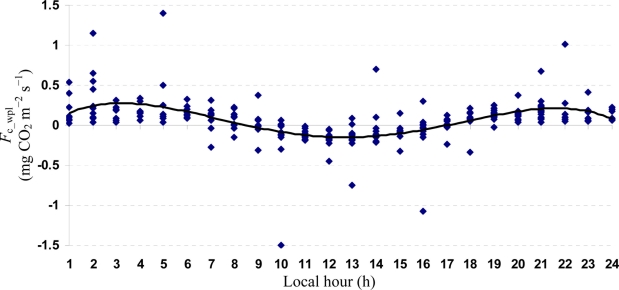
A quartic function fitted to *F*_c_wpl_ dataset for Yenicaga peatland in October of 2010: *F*_c_wpl_ (mg CO_2_ m^−2^ s^−1^) = 0.1951 h − 0. 0423 h^2^ ± 0.0027 h^3^ − 5 × 10^−5^ h^4^; (*R*^2^_adj_ = 0.3127; SE = 0.2074; *n* = 249; *P* < 0.001).

Quartic functions (an equation of fourth degree) fitted to both entire and monthly *F*_c_wpl_ datasets as a function of the explanatory temporal variable (h) provided a meaningful representation of CO_2_ fluxes, with a *R*^2^_adj_ value range of 70.9% in July to 31.3% in October (Figures 3 to 7).

### 3.2. Multitemporal Comparisons of CO_2_ Fluxes

The entire dataset of daytime and nighttime CO_2_ fluxes (*n* = 1,884) was used for a multiple comparison according to hours, nighttime *versus* daytime, and months, based on Tukey’s test following one-way ANOVA (Table 2). A comparison of hourly mean *F*_c_wpl_ values showed that the Yenicaga peatland acted as a CO_2_ sink for the daytime periods between 9:00 AM and 17:00 PM and acted as a CO_2_ source for the periods between 18:00 PM and 8:00 AM. On average, the Yenicaga peatland had minimum and maximum CO_2_ fluxes ranging from −0.05 ± 0.21 mg CO_2_ m^−2^ s^−1^ at 9:00 AM to −0.28 ± 0.18 mg CO_2_ m^−2^ s^−1^ at 12:00 PM as a CO_2_ sink and from 0.05 ± 0.12 mg CO_2_ m^−2^ s^−1^ at 18:00 PM to 0.38 ± 0.29 mg CO_2_ m^−2^ s^−1^ at 5:00 AM as a CO_2_ source, respectively (Table 2).

Mean daytime CO_2_ flux (−0.11 ± 0.22 mg CO_2_ m^−2^ s^−1^) significantly differed from mean nighttime CO_2_ flux (0.26 ± 0.23 mg CO_2_ m^−2^ s^−1^) (*P* < 0.001). All the monthly mean CO_2_ fluxes were positive (upward into the atmosphere) and varied between 0.03 ± 0.35 mg CO_2_ m^−2^ s^−1^ in July and 0.07 ± 0.25 mg CO_2_ m^−2^ s^−1^ in September. However, the monthly mean CO_2_ fluxes did not appear to significantly differ from one another (Table 2). The best-fit MNLR model was derived from the entire dataset of nighttime and daytime *F*_c_wpl_ by exploring possible interaction effects among the EC and meteorological variables as follows:

(5) $$\begin{array}{c} F_{c\_\text{wpl}}\;\left(\text{mg}\;{\text{CO}}_{2}\;m^{-2}\;s^{-1}\right)=0.001394\;\text{month}\pm 0.13776\;h-0.029586\;h^{2}\pm 0.0018587\;h^{3}-\\ 0.0000358\;h^{4}-0.05387\;\text{daytime}-0.003694\;H_{s}\pm 0.00001183\;H_{s}^{2}\pm 0.016833\;\text{log}(H_{s}^{2})-\\ 0.0008682\;{\text{LE}}_{\text{wpl}}-0.00000059\;{\text{LE}}^{2}\pm 0.038155\;\text{log}\left({\text{LE}}^{2}\right)-0.000013P_{a}-0.009471\;T_{a}-\\ 0.000879\;\text{RH }\pm 0.00022167\;{\text{RH}*T}_{a}-0.007483\;R_{s\_\text{dn}}\pm 0.0000339\;R_{s\_\text{dn}}*R_{1\_\text{up}}-\\ 0.00000001\;{\text{RH}*T}_{a}*R_{s\_\text{dn}}*R_{1\_\text{up}}\pm 0.009735\;\;U_{z}*R_{s\_\text{dn}}\\ \left(R_{\text{adj}}^{2}=62.6\%;\text{SE}=0.181;\;n=1882;\;P<0.001\right) \end{array}$$

where the variable “daytime” was used as an indicator variable coded as 1 and 0 for daytime and nighttime, respectively, and the sign “*” between the variable notations denotes two-to-four-way interactions.

### 3.3. Ecosystem Components of Carbon Metabolism

**Figure 8.**
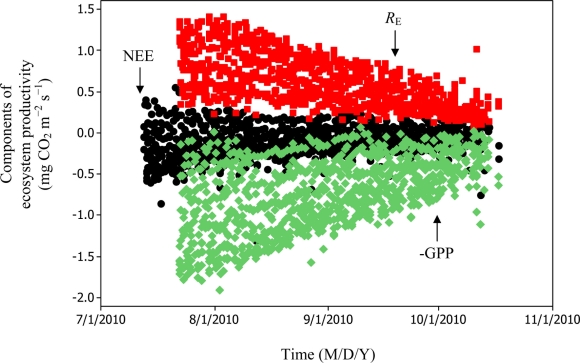
Estimation of GPP and daytime *R*_E_ components for the Yenicaga peatland based on Equation (6) (*n* = 1021 for NEE; and *n* = 896 for GPP and *R*_E_).

**Figure 9.**
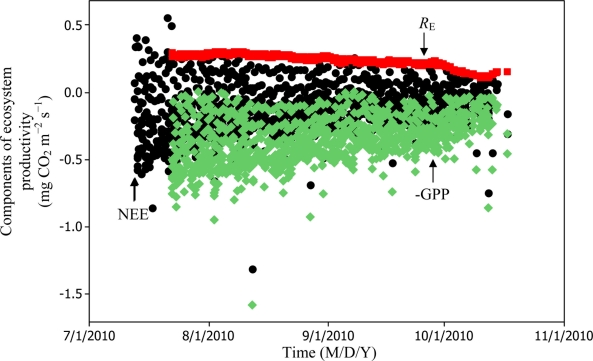
Estimation of GPP and daytime *R*_E_ components for the Yenicaga peatland based on Equation (11) (*n* = 1021 for NEE; and *n* = 896 for GPP and *R*_E_).

Nighttime *R*_E_ was modeled using (1) best-fit M(N)LR models with/without the forced inclusion of temporal variables (hour and month), and (2) ST-dependent *R*_soil_ equations [Equations (1) and (2)] of RothC and CENTURY models with/without the forced addition of SWC. All the *R*_E_ models were built with the intercept forced through zero as follows:

(6) $$\begin{array}{c} R_{E}\;(\text{mg}\;{\text{CO}}_{2}\;m^{-2}\;s^{-1})=0.0004922\;h-0.02111\;\text{month}\pm 0.13747\;T_{\text{sonic}}-0.1496\;T_{a}\pm 0.01119\\ {\text{ST}}_{\text{min}}\pm 0.0032329\;\text{SWC}\pm 0.5456\;u*\pm \;0.5201\;U_{z}-0.04903\;\text{WS}\;-0.00061\;P_{a}\pm 0.0021203\;R_{n}\\ -0.0027481\;H_{s}\\ \left(R_{\text{adj}}^{2}=73.8\%;\text{SE}=0.0473;n=203;P<0.001\right) \end{array}$$

(7) $$\begin{array}{c} R_{E}\;(\text{mg}\;{\text{CO}}_{2}\;m^{-2}\;s^{-1})=0.005383\;T_{\text{sonic}}\pm 0.0026921\;\text{SWC}\pm 1.0846\;u*\pm \;0.0733\;U_{z}-0.09595\\ \text{WS}\pm 0.0009581\;H_{s}-0.0003665\;\text{RH}\\ \left(R_{\text{adj}}^{2}=70.8\%;\text{SE}=0.0677;\;n=207;P<0.001\right) \end{array}$$

(8) $$\begin{array}{c} R_{E}\;(\text{mg}\;{\text{CO}}_{2}\;m^{-2}\;s^{-1})=0.33084\;\text{CENTURY}\\ \left(R_{\text{adj}}^{2}=42.0\%;\text{SE}=0.0708;\;n=207;P<0.001\right) \end{array}$$

(9) $$\begin{array}{c} R_{E}\;(\text{mg}\;{\text{CO}}_{2}\;m^{-2}\;s^{-1})=0.085842\;\text{RothC}\\ \left(R_{\text{adj}}^{2}=44.3\%;\text{SE}=0.06868;n=207;P<0.001\right) \end{array}$$

(10) $$\begin{array}{c} R_{E}\;(\text{mg}\;{\text{CO}}_{2}\;m^{-2}\;s^{-1})=0.071387\;\text{RothC}\pm 0.0005546\;\text{SWC}\\ \left(R_{\text{adj}}^{2}=53.0\%;\text{SE}=0.0684;n=207;P<0.001\right) \end{array}$$

(11) $$\begin{array}{c} R_{E}\;(\text{mg}\;{\text{CO}}_{2}\;m^{-2}\;s^{-1})=0.28185\;\text{CENTURY}\pm 0.0004839\;\text{SWC}\\ \left(R_{\text{adj}}^{2}=53.3\%;\;\text{SE}\;=0.0708;\;n=207;P<0.001\right) \end{array}$$

where *T*_sonic_ is sonic temperature (°C), *U*_z_ is vertical wind speed (m s^−1^), and *P*_a_ is air pressure (kPa). Nighttime *R*_E_ Equations (6) and (11) were used to estimate daytime RE values which in turn led to the estimation of −GPP component from Equation (3) as can be seen in Figures 8 and 9.

**Figure 10.**
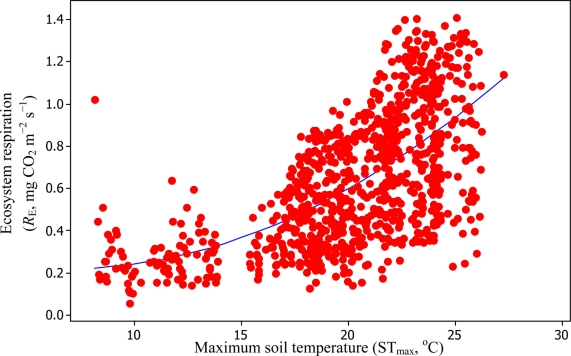
A comparison of ST_max_ *versus* daytime *R*_E_ based on Equation (6) for the Yenicaga peatland (*R*^2^_adj_ = 39.3%; SE = 0.245; *n* = 896; *P* < 0.001).

**Figure 11.**
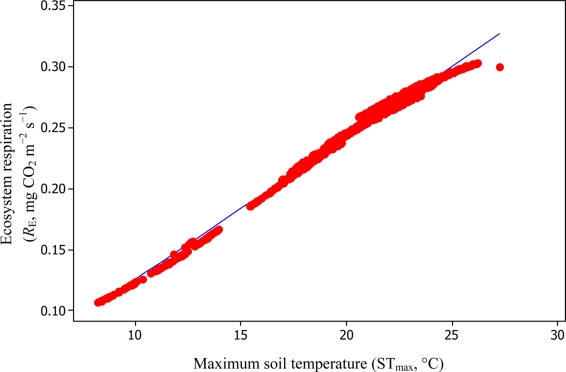
A comparison of ST_max_ *versus* daytime *R*_E_ based on Equation (11) for the Yenicaga peatland (*R*^2^_adj_ = 99.3%; SE = 0.0039; *n* = 896; *P* < 0.001).

A comparison of daytime *R*_E_ *versus* ST_max_ resulted in *R*^2^_adj_ values of 39.3% and 99.3% based on Equations (6) and (11), respectively (Figures 10 and 11).

**Figure 12.**
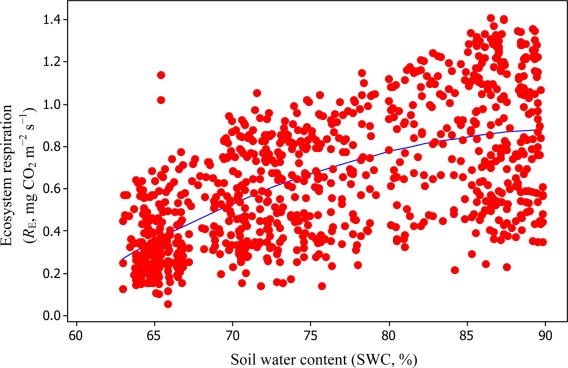
A comparison of SWC *versus* daytime *R*_E_ based on Equation (6) for the Yenicaga peatland (*R*^2^_adj_ = 39.2%; SE = 0.245; *n* = 896; *P* < 0.001).

**Figure 13.**
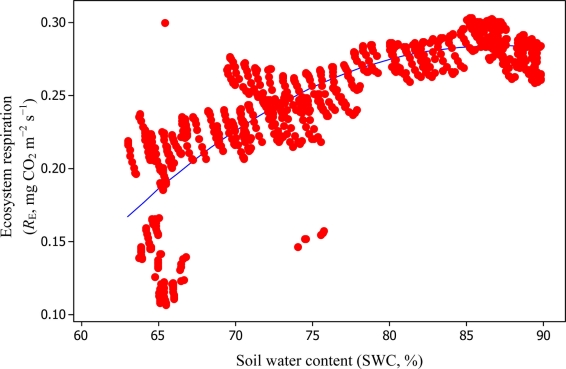
A comparison of SWC *versus* daytime *R*_E_ based on Equation (11) for the Yenicaga peatland (*R*^2^_adj_ = 68.7%; SE = 0.025; *n* = 896; *P* < 0.001).

**Figure 14.**
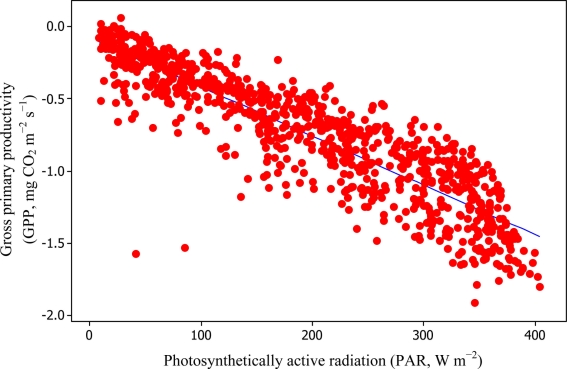
Quantification of relationship between PAR and GPP based on Equation (6) for the Yenicaga peatland (*R*^2^_adj_ = 80.3%; SE = 0.1929; *n* = 896; *P* < 0.001).

**Figure 15.**
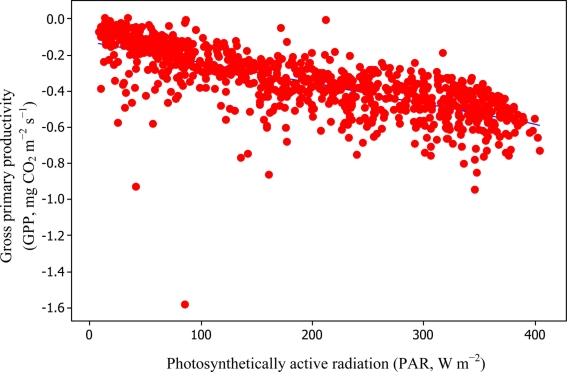
Quantification of relationship between PAR and GPP based on Equation (11) for the Yenicaga peatland (*R*^2^_adj_ = 55.0%; SE = 0.1182; *n* = 896; *P* < 0.001).

Soil water content was also determined to play a significant role in controlling daytime *R*_E_ from the Yenicaga peatland. As a function of SWC, quadratic regression models elucidated 39.2% and 68.7% of variation in daytime *R*_E_ according to Equations (6) and (11), respectively (Figures 12 and 13). A significant relationship between estimated GPP and PAR values was found, yielding *R*^2^_adj_ values of 80.3% and 55% from Equations (6) and (11), respectively (Figures 14 and 15). The rate of NEE in the Yenicaga peatland for the study period was on average estimated at 0.11 mg CO_2_ m^−2^ s^−1^ (26.5 kg C ha^−1^ day^−1^) and ranged from −1.26 mg CO_2_ m^−2^ s^−1^ (−297 kg C ha^−1^ day^−1^) to 1.06 mg CO_2_ m^−2^ s^−1^ (249 kg C ha^−1^ day^−1^). This case points to the domination of total (positive daytime ± nighttime fluxes) *R*_E_ over GPP (negative flux) throughout most of the study period, especially, towards the end of the period during which GPP decreased at a faster rate than *R*_E_ (Figures 8 and 9). During the peak growing season in July, GPP peaked and decreased as the season progressed. Over the study period, GPP was on average −0.74 ± 0.43 mg CO_2_ m^−2^ s^−1^ (−175 ± 102 kg C ha^−1^ day^−1^) and −0.35 ± 0.18 mg CO_2_ m^−2^ s^−1^ (−2 ± 41 kg C ha^−1^ day^−1^) according to Equations (6) and (11), respectively. On average, nighttime *R*_E_ was estimated at 0.23 ± 0.09 mg CO_2_ m^−2^ s^−1^ (53 ± 22 kg C ha^−1^ day^−1^) based on the EC sensors, while daytime *R*_E_ values were 0.64 ± 0.31 mg CO_2_ m^−2^ s^−1^ (150 ± 74 kg C ha^−1^ day^−1^) and 0.24 ± 0.05 mg CO_2_ m^−2^ s^−1^ (57 ± 11 kg C ha^−1^ day^−1^) based on Equations (6) and (11), respectively.

Peatlands (bog hummock, bog hallow, and poor fen) in a cool temperate region of eastern Canada were reported to have NEE range from −0.18 to 0.13 mg CO_2_ m^−2^ s^−1^; nighttime *R*_E_ range from 0.07 to 0.36 mg CO_2_ m^−2^ s^−1^; and GPP range from −0.20 to −0.33 mg CO_2_ m^−2^ s^−1^ [15,16]. The reported ranges of NEE, *R*_E_ and GPP values are in a close agreement with our findings for the Yenicaga peatland [NEE = 0.11 mg CO_2_ m^−2^ s^−1^ based on the EC data; nighttime *R*_E_ = 0.23 mg CO_2_ m^−2^ s^−1^ based on the EC data; daytime *R*_E_ = 0.24 mg CO_2_ m^−2^ s^−1^ based on Equation (11); daytime *R*_E_ = 0.64 mg CO_2_ m^−2^ s^−1^ based on Equation (6); GPP = −0.35 mg CO_2_ m^−2^ s^−1^ based on Equation (11); and GPP = −0.74 mg CO_2_ m^−2^ s^−1^ based on Equation (6)], in particular, based on Equation (11). Similarly, EC measurements from 12 wetlands ranging from ombrotrophic and minerotrophic peatlands to wet tundra ecosystems across Europe and North America under temperate-to-arctic climate regimes showed that CO_2_ fluxes in July varied considerably between −3 and 1 g C m^−2^ day^−1^ for NEE; 1 and 4 g C m^−2^ day^−1^ for *R*_E_; and −1 and −6 g C m^−2^ day^−1^ for GPP [17], which were close to our results.

## 4. Conclusions

Carbon metabolism components of the Yenicaga peatland as measured by EC sensors and quantified by M(N)LR models clearly revealed that diurnal and seasonal variations in exchange rates of CO_2_ between the atmosphere and the peatland ecosystem are dependent on the magnitude, rate and timing of GPP and total *R*_E_, which are in turn strongly controlled by the dynamics of soil moisture and temperature, and PAR. Peatlands experiencing drought conditions are reported to be able to act as a CO_2_ source. Given the total PPT: ET ratio of 0.3 during the study period, the Yenicaga peatland is considered to undergo a dry season and signals what future rate and amount of changes may be expected in the face of an increase in ET, air temperature and ecosystem disturbances as well as a decrease in PPT. The present study is the first one to quantify CO_2_ exchanges for a peatland in Turkey using real-time monitoring by the EC sensors. It is also the first time in Turkey that diurnal source and sink behaviors of a rarely occurring ecosystem like the Yenicaga peatland, which at the same time undergoes severe human-induced pressures such as conversions to rangeland and cropland, and peat mining, were quantitatively assessed using EC sensors. Further research is needed to explore an integration of remote and proximal sensors, and biogeochemical process-based models in improving our understanding and predicting impacts of human-induced disturbances on ecosystem metabolism.

## Figures and Tables

**Table 1.** Descriptive statistics of eddy covariance and meteorological data collected on an hourly basis in the Yenicaga peatland.

**Variable**	***n***	**mean**	**±SD**	**CV**	**min**	**median**	**max**
*F*_c_wpl_ (mg CO_2_ m^−2^ s^−1^)	1,884	0.06	0.30	498	−1.50	0.06	1.53
*H*_s_(W m^−2^)	1,884	38.40	58.44	152	−78.86	3.01	238.80
LE_wpl_ (W m^−2^)	1,884	102.69	130.79	127	−92.89	41.40	517.29
u* (m s^−1^)	1,884	0.18	0.12	68	0.01	0.14	0.57
CO_2_ density (mg m^−3^)	1,884	633.34	118.19	19	243.45	598.57	1,577.23
H_2_O density (g m^−3^)	1,884	12.34	4.77	39	−2.62	12.94	47.82
*T*_a_ (°C)	1,884	18.15	7.05	39	−0.67	17.95	34.00
RH (%)	1,884	71.54	22.30	31	16.09	76.13	107.80
WD (degree)	1,884	219.91	127.29	58	0.04	265.93	359.89
WS (m s^−1^)	1,884	1.73	1.40	81	0.04	1.19	6.14
*R*_s_up_ (W m^−2^)	1,884	263.80	309.91	117	−4.91	104.18	981.88
*R*_s_dn_ (W m^−2^)	1,884	44.14	48.88	111	−0.19	18.63	153.55
*R*_l_up_ (W m^−2^)	1,884	386.85	34.95	9	294.45	386.94	474.95
*R*_l_dn_ (W m^−2^)	1,884	413.84	47.55	11	307.63	405.96	531.50
*R*_n_ (W m^−2^)	1,884	192.66	245.44	127	−35.80	68.69	766.03
ET (mm h^−1^)	1,680	0.14	0.21	124	0.0	0.02	0.75
PPT (mm h^−1^)	1,680	0.04	0.36	825	0.0	0.0	7.8
ST_min_ (°C)	1,680	19.66	3.92	20	8.11	20.03	26.18
ST_max_ (°C)	1,680	19.84	3.96	20	8.19	20.24	27.24
SWC (%)	1,680	75.37	8.73	12	62.98	73.75	89.90

F_c_wpl_ = WPL-corrected CO_2_ flux; *H*_s_ = sensible heat flux; LE_wpl_ = WPL-corrected latent heat flux; u* = friction velocity; *T*_a_ = air temperature; RH = relative humidity; WD = wind direction; WS = wind speed; *R*_s_up_ = upwelling shortwave radiation; *R*_s_dn_ = downwelling shortwave radiation; *R*_l_up_ = upwelling longwave radiation; *R*_l_dn_ = downwelling longwave radiation; *R*_n_ = net radiation; ET = evapotranspiration; ST_min_ = minimum soil temperature; ST_max_ = maximum soil temperature; SWC = soil water content; CV = coefficient of variation; and SD = standard deviation.

**Table 2.** A multiple comparison of mean *F*_c_wpl_ fluxes (mg CO_2_ m^−2^ s^−1^) in the Yenicaga peatland by different temporal scales (*P* < 0.001).

**Local hour**	***n***	**mean**	**SD**	**Comparison based on 95% confidence intervals**
1:00	57	0.254	0.248	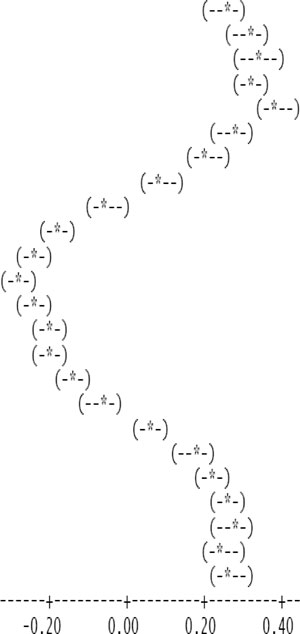
2:00	53	0.313	0.242	
3:00	55	0.337	0.277	
4:00	69	0.320	0.229	
5:00	65	0.382	0.296	
6:00	72	0.276	0.215	
7:00	85	0.209	0.256	
8:00	76	0.087	0.295	
9:00	82	−0.053	0.210	
10:00	90	−0.171	0.187	
11:00	89	−0.242	0.203	
12:00	91	−0.280	0.187	
13:00	91	−0.237	0.122	
14:00	91	−0.191	0.168	
15:00	89	−0.200	0.156	
16:00	88	−0.144	0.158	
17:00	90	−0.069	0.101	
18:00	91	0.052	0.128	
19:00	89	0.171	0.145	
20:00	91	0.222	0.117	
21:00	85	0.262	0.149	
22:00	69	0.275	0.193	
23:00	72	0.248	0.212	
24:00	54	0.267	0.266	

**Day *vs.* night**	*n*	mean	SD	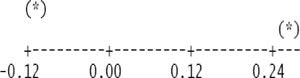

Daytime	1030	−0.111	0.222	
Nighttime	852	0.266	0.235	

**Month**	*n*	mean	SD	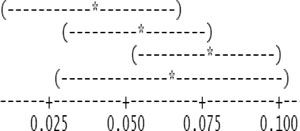

July	412	0.039	0.351	
August	620	0.054	0.307	
September	603	0.077	0.257	
October	249	0.064	0.248	
